# Partial Replacement of Oat Hay with Whole-Plant Hydroponic Barley Seedlings Modulates Ruminal Microbiota and Affects Growth Performance of Holstein Heifers

**DOI:** 10.3390/microorganisms10102000

**Published:** 2022-10-10

**Authors:** Peng Ren, Mengmeng Deng, Juan Feng, Ruocheng Li, Xiaojiao Ma, Jianxin Liu, Diming Wang

**Affiliations:** Institute of Dairy Science, College of Animal Sciences, Zhejiang University, Hangzhou 310058, China

**Keywords:** hydroponic barley seedlings, growth performance, nutrient digestibility, nitrogen recycle, ruminal microbiota, Holstein heifer

## Abstract

The dairy industry is facing challenges in balancing forage supply and crop production. Therefore, forage supply based on a farm land-saving approach should be developed to overcome the human–livestock competition on farmland. The objective of this study was to learn the potential impact of partially replacing oat hay with whole-plant hydroponic barley seedlings (HBS) produced via a land-saving hydroponic method on growth performance, digestibility, and rumen microbiota in Holstein dairy heifers. In total, 39 Holstein heifers were randomly divided into 13 blocks based on age and body weight for an 8-week experimental period. The heifers within each block were randomly allocated to one of three diets group: (1) 0% HBS and 16% oat hay (CON); (2) 4% HBS and 12% oat hay (25% HBS); and (3) 8% HBS and 8% oat hay (50% HBS). Compared to CON, feed intake, growth performance, and body N retention were similar to those in cows fed 25% HBS but lower in 50% HBS-fed animals (*p <* 0.05). Reduced digestibility (crude protein (CP) and organic matter (OM)) was observed in 50% HBS animals (*p <* 0.05). Compared to the control, the levels of *Lachnospiraceae_XPB1014_group*, *Bacillus,* and *Colidextribacter* were higher, but the levels of *Sphaerochaeta* and *Ruminiclostridium* were lower in 50% HBS animals (*p <* 0.05). Additionally, the digestibility of CP (*p <* 0.01, r = −0.61) and ether extract (EE) (*p <* 0.01, r = −0.58) was negatively correlated with *Lachnospiraceae_XPB1014_group*. The digestibility of OM (*p =* 0.01, r = −0.55), neutral detergent fiber (NDF) (*p =* 0.01, r = −0.56), acid detergent fiber (ADF) (*p =* 0.02, r = −0.52), and CP (*p <* 0.01, r = −0.61) was negatively correlated with *Bacillus*. The digestibility of NDF (*p =* 0.02, r = −0.52) and ADF (*p =* 0.03, r = −0.50) was negatively correlated with *Colidextribacter*. The digestibility of OM (*p =* 0.03, r = 0.50), NDF (*p =* 0.03, r = 0.50), and ADF (*p =* 0.03, r = 0.49) was positively correlated with *Ruminiclostridium*. The digestibility of OM (*p =* 0.04, r = 0.47), CP (*p <* 0.01, r = 0.58), and EE (*p =* 0.03, r = 0.49) was positively correlated with *unclassified_f_Rikenellaceae*. The digestibility of CP was positively correlated with *Sphaerochaeta* (*p =* 0.02, r = 0.53). In conclusion, the current study suggests that HBS could replace oat hay in a ratio-dependent manner. The reduced growth performance could be caused by lower feed intake and digestibility, which may be attributed to the alteration in the rumen’s microbial population. Further exploration of the inhibiting factors of HBS would broaden the application of hydroponic feed in the future.

## 1. Introduction

Monitoring data from the Ministry of Agriculture and Rural Affairs of the People’s Republic of China (http://www.moa.gov.cn/; accessed on 23 March 2022) show that the national stock of Holstein cows was 5.612 million at the end of 2021, with an increase of 10.9% year-on-year, and this stock has maintained rapid growth for two consecutive years. The rapidly growing dairy industry has accelerated forage demand, especially in developed countries such as China. However, according to the statistics from the General Administration of Customs of the People’s Republic of China (http://www.customs.gov.cn/; accessed on 23 March 2022), 22,600 tons of oat hay was imported from January to February 2022, with a year-on-year decrease of 69.1% due to the increased cost (a year-on-year increase as high as 18.9%). Moreover, severe competition between plant-source food and animal feed impacted farmland usage and commodity prices. To save farmland for plant-sourced food production, a novel approach should be developed for feed supply, and especially for forage supply, to maintain the sustainable development of the dairy industry.

Hydroponics is a soil-free plant-culture method that has specific temperature and humidity requirements and that requires an ecologically controlled environment [1]. A recent study suggests hydroponic systems can be used to guarantee the constant production of a high quantity of green forage for livestock throughout the year at acceptable prices [2]. Moreover, plants grow rapidly in hydroponic systems, with hydroponic fodder with interwoven roots growing as high as 20 to 30 cm within a short period of time (7 to 10 days) [2], and this is affected by the plant species and environmental index [3,4]. During sprouting, the content of organic matter (OM), dry matter (DM), and nitrogen-free extract decrease, but crude fiber (CF), NDF, ADF, acid detergent fibers, and ash increase [2]. A recent study found a 21.9% loss in DM from original seeds after sprouting for a period of 7 days [5]. The protein rate (23%, DM basis) and NDF (26.7%, DM basis) seem to represent an optimized alternative for high-quality forage, such as alfalfa and oat hay [4].

Some studies have been conducted to investigate the potential application of hydroponic green fodder production for livestock. For example, the digestibility of CP and CF was enhanced in cows fed hydroponic maize fodder, leading to a 13.7% increase in milk yield compared to cows fed conventional Napier–Bajra hybrid green fodder [6]. DMI, the digestibility of nutrients, and growth rate were higher in male Hariana calves after replacing the concentrate mixture and conventional green fodder with hydroponic barley fodder [7]. Compared to CON, growing goats experienced higher total DM intake, feed conversion efficiency, and body weight gain when fed finger millet straw, hydroponically grown maize, and hydroponic barley fodder (60:40:0 or 60:20:20) [3]. However, the DMI, net nutrient availability, and growth performance were lower in lambs fed a diet that replaced mixture/conventional green fodder with maize grain sprouts [8]. The inconsistent results obtained from different studies reveal a knowledge gap in the digestion characteristics of hydroponic fodder.

A few studies have reported on the interaction between hydroponic substrates and animal gut microbiota, we hypothesize that HBS can affect nutrient metabolism by modulating the relative abundance of rumen microbiota, thereby affecting the growth performance of heifers. Therefore, the present study was conducted to investigate how a ratio-dependent replacement of HBS for oat hay impacts the growth performance and digestibility of Holstein heifers through interaction with rumen microbiota.

## 2. Materials and Methods

### 2.1. Hydroponic Barley Seedling Production

Hydroponic barley seedlings were produced in a hydroponic fodder production unit measuring 12.2 × 2.4 × 2.9 m in length, width, and height, respectively. Semi-automated irrigating sprayers were used to irrigate the barley grains with tap water without any nutrient supplementation [9]. Tap water was used to the irrigate barley grains twelve times each day: at 0:00, 2:00, 4:00, 6:00, 8:00, 10:00, 12:00, 14:00, 16:00, 18:00, 20:00, and 22:00, for 25 s each time. The temperature of the planting room was controlled to maintain a temperature range of 18–20 °C and relative humidity of 70–90%. LED plant lamps with waterproof devices were placed vertically on the walls to promote the growth of leaves. About 2 kg of barley seeds were placed in nylon net bags and soaked in 1.6% sodium hypochlorite solution for disinfection for 1 h. Then, they were rinsed with clean water 5 times to remove the disinfectant, and finally, they were soaked in cold water overnight. The following morning, the seeds were removed from the water and were taken out of the bags, and laid on the planting plate. The growth cycle of barley seedings is 7 d, where days 1 to 2 are the germination period, during which the seeds are grown in a dark area, and days 3 to 7 are the aboveground growth period. On the third day, it was necessary to push the planting plate with the seeds on it into a light area, where a light–dark cycle of illumination for 18 h and darkness for 6 h was maintained and where the photosynthetic photon flux density (PPFD) was 50–100 μmol/m^2^/s. The leaf height was 10~23 cm after growing for 7 d.

### 2.2. Nutritional Compositions of Hydroponic Barley Seedlings

About 1 kg of the fresh HBS was cut and mixed thoroughly and then dried in a precision blast drying oven (BAO-150A, STIK Co., Ltd., Shanghai, China) at 105 °C for 15 min to inactivate the enzymes. Then, the HBS were dried at 65 °C for 48 h, ground through a 1 mm screen in a water mill commonly used in traditional Chinese medicine (HK-08B, Xulang mechanical equipment Co., Ltd., Guangzhou, China), preserved at 4 °C, and analyzed following using the same methods as those used for feed. In our study, the HBS had 12.70% DM, 22.43% CP, 6.22% EE, 45.84% NDF, 23.05% ADF, 4.11% ash (DM basis), and 5.35 NE_L_ (MJ/kg DM).

### 2.3. Animals and Experimental Design

Animal use and care were approved by the Animal Care Committee of Zhejiang University (Hangzhou, China). The feeding experiment was conducted at the Zhengxing Dairy Farm (Hangzhou, China). In total, 39 Holstein heifers were randomly divided into 13 blocks (3 groups) based on age (253 ± 37.39 d) and body weight (283.72 ± 32.83 kg). The heifers within each block were randomly allocated to one of three treatment groups: 0% hydroponic barley seedlings (HBS) and 16% oat hay (CON), 4% HBS and 12% oat hay (25% HBS), and 8% HBS and 8% oat hay (50% HBS). The study lasted for 8 weeks after a 1-week adaptation period. During the adaptation period, the 25% HBS group had oat hay replaced at a rate of 5% on the first day, and the rate increased by 5% every day. It reached 25% replacement on the fifth day, and the 25% replacement was maintained throughout the rest of the study. Additionally, the 50% HBS group had oat hay replaced by 5% on the first day and by 10% on the second day. From then on, the replacement ratio increased by 10% every day to 50% on the sixth day, and the 50% replacement was maintained throughout the study. The heifers were housed in individual stall barns and had free access to water and were fed twice daily (0630 and 1630 h). The three diets were fed as a TMR and were offered ad libitum to yield 5% to 10% orts.

### 2.4. Sample Collection and Analysis

The diets offered and refusals were weighed and recorded for 3 consecutive days at 0, 4, and 8 weeks to determine the dry matter intake (DMI). Samples of the diets were collected daily during the experimental period, and the samples were dried at 65 °C for 48 h in a precision blast drying oven (BAO-150A, STIK Co., Ltd., Shanghai, China). The dried samples were ground through a 1 mm screen in a water mill commonly used in traditional Chinese medicine (HK-08B, Xulang mechanical equipment Co., Ltd., Guangzhou, China) and were preserved at 4 °C. Subsequently, the samples were analyzed for dry matter (DM, 105 °C for 5 h), crude protein (CP, # 988.05), ether extract (EE, # 920.39), crude ash (ash, # 942.05), and acid detergent fiber (ADF, # 973.18) [10]. The content of NDF was determined using heat-stable alpha-amylase and sodium sulfite [11]. An ANKOM2000 fiber analyzer (Ankom Technology Corp., Macedon, NY, USA) was used to analyze the NDF and ADF concentrations. The net energy for lactation (NE_L_) was calculated via the following equation [12]: GE (MJ/kg DM) = (CP% × 5.7 + EE% × 9.4 + CF% × 4.2 + NFE% × 4.2) × 0.04184; GED (%) = 94.2808 − 61.5370 × (NDF/OM); DE (MJ/kg DM) = GE (MJ/kg DM) × GED; NE_L_ (MJ/kg DM) = 0.5501 × DE − 0.3958,where GE, NFE, GED and DE were gross energy, nitrogen-free extract, gross energy digestibility and digestible energy, respectively. The ingredients and nutrient compositions of the experimental diets are presented in Table 1.

Body weight (BW), body height, body oblique length, and chest circumference were measured using an electronic weighbridge, a measuring stick, and leather measuring tape, for 3 consecutive days before morning feeding at 0, 4, and 8 weeks. The average daily increases in body weight and feed efficiency were calculated according to the following formulae [13]: ((kg of final BW − kg of initial BW)/experimental days] and (kg of the average daily gain/kg of the DMI), respectively. The calculation method for the average daily increases in body height, body oblique length, and chest circumference was the same as for the average daily increase in BW.

Fecal samples were collected from all heifers at 06:00, 12:00, and 18:00 for 2 consecutive days at 0, 4, and 8 weeks by stimulating the rectum. The samples obtained during the two days were evenly mixed to obtain mixed fecal samples of about 500 g, and about of 50 mL, 10% hydrochloric acid was added for nitrogen fixation. The samples were treated and analyzed for CP, EE, NDF, ADF, and ash using the same methodology as used to determine the compositions of the diets. The percentage of organic matter (OM) was equal to 100 minus the percentage of ash. The content of acid-insoluble ash (AIA) in the feces and diets was measured [14]. The nutrient-apparent digestibility was determined using the AIA as a marker and was calculated via the following equation [15]:

Nutrient apparent digestibility (%) = [1 − (Ad × Nf)/(Af × Nd)] × 100, where Ad and Af are the concentrations of AIA in the diets and feces, respectively, and Nf and Nd are the concentrations of nutrient in the feces and diets, respectively.

Spot urine samples were collected for 2 consecutive days at 0, 4, and 8 weeks at 06:00, 12:00, and 18:00 by stimulating the vagina. The urine samples were pooled by individual animals, acidified with 0.036 mol/L H_2_SO_4_ at a ratio of 1:4, and were immediately stored at −20 °C for later analysis of the creatinine and urea nitrogen levels using commercial kits (Nanjing Jiancheng Bioengineering Institute, Nanjing, China) according to the manufacturer’s instructions. Creatinine was used to estimate urine volume using the following equation [16]: BW (kg) × 29 (mg/d)/creatinine concentration (mg/L).

Ruminal fluid was collected in week 8 using an oral stomach tube approximately 3h after morning feeding. The first ruminal samples of about 150 mL were discarded to avoid saliva contamination [17]. Triplicate 1 mL samples were frozen at −80 °C for later analysis of the diversity of the microbial communities using high-throughput sequencing technology. We randomly selected 7 samples from each treatment group, and a total of 21 samples were sequenced. DNA extraction, library construction, and sequencing were conducted at Shanghai Majorbio Bio-Pharm Technology Co., Ltd. (Shanghai, China). Briefly, microbial DNA was extracted using the Fast DNA^®^ Spin Kit for Soil (MP Biomedicals, Santa Ana, CA, USA) according to the manufacturer’s protocols. The DNA extract was checked with 1% agarose gel, and the DNA concentration and purity were determined with a NanoDrop 2000 UV-vis spectrophotometer (NanoDrop 2000, Thermo Scientific, Wilmington, MA, USA). The V3-V4 region of the bacteria’s 16S rRNA genes was amplified by an ABI PCR thermocycler (GeneAmp^®^ 9700,ABI, CA, USA) as follows: initial denaturation at 95 °C for 3 min followed by 27 cycles of denaturing at 95 °C for 30 s, annealing at 55 °C for 30 s and extension at 72 °C for 45 s, single extension at 72 °C for 10 min, and ending at 4 °C using the primer pairs 338F (5′-ACTCCTACGGGAGGCAGCAG-3′) and 806R (5′-GGACTACHVGGGTWTCTAAT-3′). The PCR mixtures contained 5 × *TransStart* FastPfu buffer 4 μL, 2.5 mM dNTPs 2 μL, forward primer (5 μM) 0.8 μL, reverse primer (5 μM) 0.8 μL, *TransStart* FastPfu DNA Polymerase 0.4 μL, template DNA 10 ng, and finally ddH_2_O up to 20 μL. PCR reactions were performed in triplicate. The PCR product was extracted from 2% agarose gel and purified using the AxyPrep DNA Gel Extraction Kit (Axygen Biosciences, Union City, CA, USA) according to the manufacturer’s instructions and quantified using Quantus™ Fluorometer (Promega, Madison, WI, USA).

The raw 16S rRNA gene sequencing reads were demultiplexed, quality-filtered using fastp version 0.20.0 [18], and merged using FLASH version 1.2.7 [19] with the following criteria: (i) the 300 bp reads were truncated at any site receiving an average quality score of <20 over a 50 bp sliding window, and truncated reads shorter than 50 bp were discarded. Reads containing ambiguous characteristics were also discarded; (ii) only overlapping sequences longer than 10 bp were assembled according to their overlapped sequence. The maximum mismatch ratio of overlap regions was 0.2. Reads that could not be assembled were discarded; (iii) samples were distinguished according to the barcode and primers, the sequence direction was adjusted, and exact barcode matching and 2-nucleotide mismatches were carried out during primer matching.

Operational taxonomic units (OTUs) with a 97% similarity cutoff [19,20,21] were clustered using UPARSE version 7.1 [20], and chimeric sequences were identified and removed. The taxonomy of each OTU representative sequence was analyzed using RDP Classifier version 2.2 [22] against the 16S rRNA database (e.g., Silva v138) using a confidence threshold of 0.7.

### 2.5. Statistical Analysis

Data were analyzed using SAS software (version 9.2). DMI, body size indexes, and body weight were analyzed using the PROC MIXED procedure with covariance type AR (1). A randomized block design with repeated measures was used, with treatment, week, and interaction of treatment × week as the fixed effects and the cows within the treatment as a random effect. The data obtained from week 0 were added to the model as covariates during the statistical analysis. The nutrient digestibility, nitrogen recycling, the average daily growth in body height, body oblique length and chest circumference, average dry matter intake, average daily gains, and feed efficiency were analyzed using the one-way ANOVA procedure with HBS treatment used as the main factor. Microbial diversity data was analyzed using the majorbio cloud platform (https://cloud.majorbio.com; accessed on 25 May 2022). The Kruskal–Wallis test was used to analyze the differential ruminal bacteria, and correlation analysis between the differential ruminal bacteria and nutrient digestibility were calculated using Spearman’s correlation test. Significant difference was set at *p <* 0.05, and 0.05 < *p <* 0.10 were defined as statistical trends.

## 3. Results

### 3.1. Feed intake and Growth Performance

**Figure 1:**
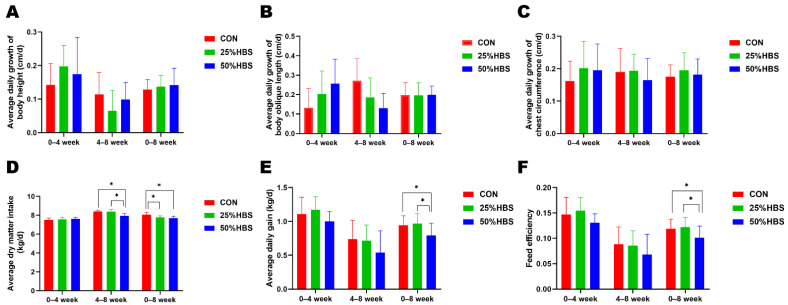
Growth performance of heifers in different treatment groups. (**A**) Average daily growth of body height. (**B**) Average daily growth of body oblique length. (**C**) Average daily growth of chest circumference. (**D**) Average dry matter intake. (**E**) Average daily gains. (**F**) Feed efficiency. Star (*) indicates that there are significant differences between groups.

As shown in Table 2, compared to the animals in CON, DMI was similar in 25% HBS animals but lower in 50% HBS-fed cows (*p <* 0.05). Body height, body oblique length, and chest circumference were similar in heifers with different diets (*p >* 0.05). Although BW was similar in 25% HBS animals, it was lower in 50% HBS-fed cows (*p <* 0.05). The average daily growth in body height, body oblique length, and chest circumference were similar in the different groups (*p >*0.05) (Figure 1A–C). The average dry matter intake was lower in the 50% HBS-fed cows in 4–8 weeks, and higher in CON in 0–8 weeks (*p <* 0.05) (Figure 1D). Throughout the whole experimental stage, the average daily gain (ADG) and feed efficiency were lower in the 50% HBS-fed cows (*p <* 0.05) (Figure 1E,F).

### 3.2. Nutrient Digestibility and Nitrogen Recycling

Nutrient digestibility in the heifers is shown in Table 3. The apparent digestibility of EE was similar across the three groups (*p >* 0.05). Compared to CON, the apparent digestibility of CP and OM were similar in the 25% HBS animals but were lower in the 50% HBS-fed cows (*p <* 0.05). Additionally, the apparent digestibility of NDF and ADF was the lowest in the 50% HBS-fed cows (*p <* 0.05).

As shown in Table 4, the nitrogen intake, the urine volume, the output of urinary nitrogen, and the urinary nitrogen proportion of N intake were similar across the three groups (*p >* 0.05). Across the three groups, the output of fecal nitrogen was the highest in the 50% HBS-fed cows (*p <* 0.05). Compared to CON, the fecal nitrogen proportion of N intake was similar in the 25% HBS animals but was higher in the 50% HBS-fed cows (*p <* 0.05). However, N retention and the proportion of N intake were lower in the 50% HBS-fed cows compared to the other two groups (*p <* 0.05).

### 3.3. Microbial Population and Its Association with Nutrient Digestibility

In terms of the microbial population, 2588, 2492, and 2489 microbial operational taxonomic units (OTUs) were identified in the cows in the CON, 25% HBS, and 50% HBS groups, respectively. Among them, 2112 OTUs were shared by the three groups, with 188, 113, and 144 unique OTUs being observed in the animals in the CON, 25% HBS, and 50% HBS groups, respectively (Figure S1A). No significant differences were found in alpha diversity indices such as the Chao1 index (Figure S1B), Shannon index (Figure S1C), or Simpson index (Figure S1D), among the three groups (*p >* 0.05).

**Figure 2:**
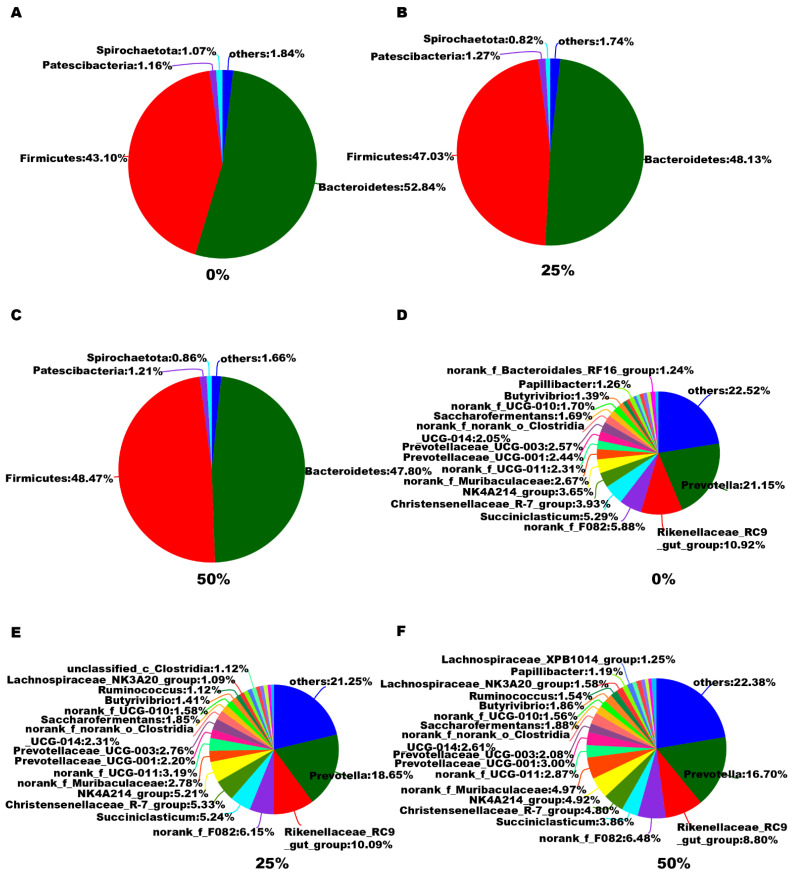
Effect of different proportions of hydroponic barley seedlings replacing oat hay on the distribution of bacterial taxa averaged under phyla (**A**–**C**) and genera (**D**–**F**) in heifers.

The relative abundance of bacterial taxa at the phylum and genus levels in heifers from the different groups is shown in Figure 2. Compared to CON, the phylum Bacteroidetes showed a tendency to decrease in abundance, and Firmicutes showed a tendency to increase in abundance in the 25% HBS- and 50% HBS-fed animals. Meanwhile, the genera *Prevotella and Rikenellaceae_RC9_gut_group* tended to decrease in the 25% HBS- and 50% HBS-fed animals. Among all of the microbial species, 15 differential bacteria had the highest genus abundance among the three groups (Table 5). Compared to CON, the mean proportions of *Lachnospiraceae_NK3A20_group* and *Agathobacter* were similar in the 25% HBS-fed animals but were greater in the 50% HBS-fed cows (*p <* 0.05). The mean proportions of *Lachnospiraceae_XPB1014_group* and *Colidextribacter* were greater in the 25% HBS- and 50% HBS-fed cows (*p <* 0.05). The mean proportion of *norank_f_Bacteroidales_RF16_group* was lower in the 25% HBS- and 50% HBS-fed cows (*p <* 0.05). The mean proportions of *unclassified_f_Lachnospiraceae* and *Bacillus* were greater in the 50% HBS-fed cows (*p <* 0.05). The mean proportions of SP3-e08, *unclassified_f_Rikenellaceae,* and *Ruminiclostridium* were lower in the 50% HBS-fed cows (*p <* 0.05). The mean proportions of *norank_f_norank_o_WCHB1-41*, *Sphaerochaeta,* and *unclassified_o_Oscillospirales* were similar in the 25% HBS-fed animals but were lower in the 50% HBS-fed cows (*p <* 0.05). The mean proportion of *Lachnospiraceae_FCS020_group* was similar in the 25% HBS- and 50% HBS-fed cows compared to in the CON cows but greater in the 50% HBS-fed cows than in the 25% HBS-fed cows (*p <* 0.05). The mean proportion of *norank_f_norank_o_LD1-PB3* was similar in the 50% HBS-fed cows but greater in the 25% HBS-fed cows (*p <* 0.05).

**Figure 3:**
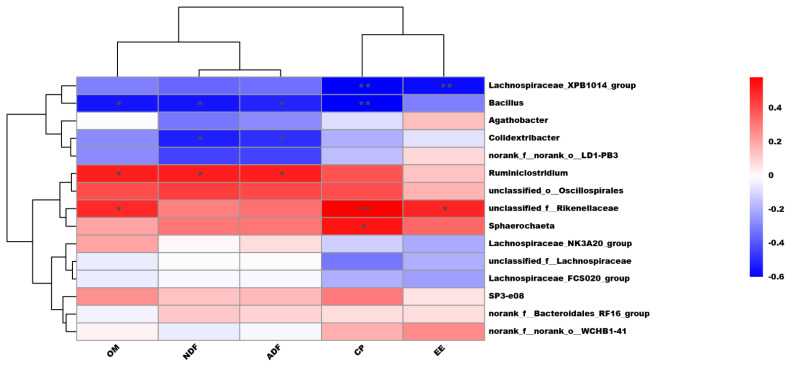
Correlation matrix between the differential ruminal bacterial genera and nutrient digestibility. * 0.01 < *p <* 0.05, ** *p <* 0.01.

Correlation analysis between the ruminal microbial profiles and nutrient digestibility is presented in Figure 3. The apparent digestibility of CP (*p <* 0.01, r = −0.61) and EE (*p <* 0.01, r = −0.58) was negatively correlated with *Lachnospiraceae_XPB1014_group*. The apparent digestibility of OM (*p =* 0.01, r = −0.55), NDF *(p =* 0.01, r = −0.56), ADF (*p =* 0.02, r = −0.52), and CP (*p <* 0.01, r = −0.61) was negatively correlated with *Bacillus*. The apparent digestibility of NDF (*p =* 0.02, r = −0.52) and ADF (*p =* 0.03, r = −0.50) was negatively correlated with *Colidextribacter*. The apparent digestibility of OM (*p =* 0.03, r = 0.50), NDF (*p =* 0.03, r = 0.50), and ADF (*p =* 0.03, r = 0.49) were positively correlated with *Ruminiclostridium*. The apparent digestibility of OM (*p =* 0.04, r = 0.47), CP (*p <* 0.01, r = 0.58), and EE (*p =* 0.03, r = 0.49) was positively correlated with *unclassified_f_Rikenellaceae*. The apparent digestibility of CP was positively correlated with *Sphaerochaeta* (*p =* 0.02, r = 0.53).

## 4. Discussion

In the present study, The DMI was similar in the 25% HBS-fed cows, but lower in the 50% HBS-fed cows compared to the CON cows. The total means of DMI in calves fed hydroponic barley green fodder was significantly lower than in those fed the control diet [23], similar to our findings. In our study, we found that a small proportion of the non-germinated seeds that attached with sprouted barely was unpalatable (Figure S2, with a germination rate of seeds around 70%), which could be an important cause of the reduced DMI in cows fed 50% HBS [9]. Moreover, as roots in the HBS lead to lower chewability and palatability, the proportion of roots in the HBS used in the current study could be the cause of the reduced feed intake in the 50% HBS-fed animals [5,24]. In addition, the reduced DMI in the 50% HBS-fed animals partially contributed to the reduced BW, average daily gain (ADG), and feed efficiency throughout the entire 8-week experimental period. Generally, growth performance is associated with nutrient digestibility.

In the current study, the digestibility of CP and OM was higher in the cows fed the CON and 25% HBS diets, and the digestibility of NDF and ADF was the lowest in the 50% HBS-fed cows. Previous studies suggest that diets containing low levels of hydroponic barley fodder improve the ability of Deccani sheep to digest OM and CP, but not NDF and ADF [25]. The digestibility of OM and CP in lambs fed a high ratio of hydroponic maize grain sprouts was comparable to the control group, and the digestibility of NDF and ADF was similar between the control and treatment groups [8]. Digestibility is affected by many factors, such as animal variety and physiological stage as well as feed variety, composition, and structure [6,7,8,25], and this could be the cause for the inconsistency of our findings with the above-mentioned studies. Nitrogen retention was not altered when freshly sprouted barley was supplemented with poor-quality roughages in sheep diets [5]. In our study, a similar nitrogen retention was found in the dairy heifers fed with the CON and 25% HBS diets, and the nitrogen retention of all the animals remained positive, suggesting that the nitrogen supply met the maintenance requirements. However, N retention and the ratio of N retention to total N intake were lower in the 50% HBS-fed cows compared to the animals in the CON and 25% HBS groups, which was consistent with the alteration in the apparent digestibility of CP in the different groups. The China Feed Data Base Information Network Center (http://www.chinafeeddata.org.cn; accessed on 13 September 2022) records show that compared to oat hay, alfalfa hay has a higher content of acid-detergent insoluble crude protein (ADICP), which is an undegradable protein that cannot be digested and utilized by ruminants [26]. A recent study reported that sprouted barley fodder had a higher content of ADICP than alfalfa hay [27], so we conjectured that HBS might have a higher content of ADICP than oat hay, affecting the digestion and absorption of protein by livestock. This may be an important reason for the lower digestibility of CP and N retention in the 50% HBS-fed cows. However, the use of hydroponic feed for ruminants, especially HBS, is still in its infancy, and we need to explore it further.

As we all know, nutrient digestibility is associated with rumen fermentation status as well as with the diversity of rumen microbiota [28]. In this study, Bacteroidetes and Firmicutes were the two dominant ruminal bacterial phyla, which is consistent with previous results [29]. We found that HBS might affect the digestibility of NDF and ADF by modulating specific ruminal microbiota related to fiber digestion. The abundance of Bacteroidetes, which plays predominant roles in fiber degradation [30], showed a tendency to decrease in the 25% HBS-fed cows and 50% HBS-fed cows. The abundance of *Prevotella and Rikenellaceae_RC9_gut_group*, members of Bacteroidetes, also showed a decreased tendency in the 25% HBS-fed cows and 50% HBS-fed cows. As we all know, *Prevotella* plays a key role in the degradation of cellulose and xylans [31], and *Rikenellaceae_RC9_gut_group* is active against soluble polysaccharides and insoluble cellulose [32]. The lower digestibility of NDF and ADF could be associated with the changes in these rumen microbial communities in the animals that consumed HBS relative to the control. Patients fed konjaku flour show an elevated abundance of *Lachnospiraceae* and *Bacillaceae* in the gut as well as a reduced body mass index and lower fat mass and percentage body fat [33]. This observation is consistent with our findings that the *Lachnospiraceae_XPB1014_group* (belonging to *Lachnospiraceae*) and *Bacillus* (belonging to *Bacillaceae*) were more highly enriched in the 50% HBS-fed cows, who showed lower BW and lower nutrient digestibility (CP, OM, NDF, ADF, and CP). A recent study reported that acetate and propionate, produced by NDF/ADF digestion, were negatively correlated with *Colidextribacter* [34], similar to our findings. Our observations are consistent with previous studies that revealed that the feces of sows with high reproductive performance contained a high abundance of *Sphaerochaeta* [35,36], and mice with higher BW had a higher abundance of *Ruminiclostridium* in their gut [37]. The development of microbial ecology-based regulation strategies may extend the potential applications of HBS in ruminants.

## 5. Conclusions

Our results revealed that a low replacement rate of oat hay with HBS maintained growth performance and feed efficiency. However, the replacement of oat hay with HBS at a high rate led to reduced ADG and feed efficiency by reducing the nutrient digestibility and N retention of dairy heifers, which is associated with alterations in the rumen microbial population. Further explorations on nutrition-digestion inhibitors of HBS would facilitate the utilization of HBS in livestock, which would be beneficial to the application of hydroponic feed.

## Figures and Tables

**Table 1:** Ingredients and nutrient compositions of the experimental diets.

Items	Treatment		
	CON	25% HBS	50% HBS
Ingredients, % DM			
Corn	2.59	2.59	2.59
Soybean meal	12.58	12.58	12.58
Wheat	2.96	2.96	2.96
DDGS ^1^	11.1	11.1	11.1
Wheat bran	5.55	5.55	5.55
Corn silage	20	20	20
Wheat stalk	27	27	27
Hydroponic barley seedlings	0	4	8
Oat hay	16	12	8
Calcium bicarbonate	0.37	0.37	0.37
Salt	0.37	0.37	0.37
Limestone powder	0.74	0.74	0.74
2% Premix ^2^	0.74	0.74	0.74
Total	100	100	100
Nutrient composition			
DM, %	47.27	45.95	45.06
CP, %DM	13.51	13.87	14.02
EE, %DM	2.13	2.25	2.41
NDF, %DM	45.99	43.97	43.48
ADF, %DM	27.26	25.30	25.13
Ash, %DM	13.09	13.40	14.11
NE_L_ ^3^, MJ/kg DM	3.98	4.10	4.08

^1^ DDGS, dried distiller grains with solubles. ^2^ Per kg of 2% premix with the following: 250,000 IU of vitamin A, 60,000 IU of vitamin D3, 1200 IU of vitamin E, 3000 mg of Zn, 630 mg of Mn, 1200 mg of Fe, 650 mg of Cu, 36 mg of I, 8 mg of Co, and 19 mg of Se. ^3^ NE_L_ was a calculated value, while nutrient composition was a measured value.

**Table 2:** Effect of different proportions of hydroponic barley seedlings replacing oat hay on body size indexes and body weight of heifers.

Items	Treatment			SEM	*p*-Value		
	CON	25% HBS	50% HBS		Treat	Week	Treat × Week
Dry matter intake, kg/d	7.99 ^a^	7.90 ^ab^	7.74 ^b^	0.06	0.04	<0.01	<0.01
Body height	120.43	121.72	121.26	0.50	0.17	<0.01	0.36
Body oblique length	129.93	131.80	132.96	0.91	0.12	<0.01	0.09
Chest circumference	155.49	157.22	156.72	0.50	0.05	<0.01	0.73
Body weight	333.12 ^a^	335.14 ^a^	326.88 ^b^	1.70	<0.01	<0.01	0.22

The values with different small letters in the same row show differences (*p <* 0.05). Values without letters indicate that no differences were observed (*p >* 0.05).

**Table 3:** Effects of different proportions of hydroponic barley seedlings replacing oat hay on nutrient digestibility of heifers.

Items	Treatment			SEM	*p*-Value
	CON	25% HBS	50% HBS		
CP	62.81 ^a^	60.26 ^a^	54.84 ^b^	0.77	<0.01
EE	75.20	72.19	71.41	0.94	0.22
NDF	51.95 ^a^	44.22 ^b^	36.84 ^c^	1.34	<0.01
ADF	49.74 ^a^	40.62 ^b^	32.58 ^c^	1.43	<0.01
OM	63.64 ^a^	60.60 ^a^	56.19 ^b^	0.78	<0.01

The values with different small letters in the same row show differences (*p <* 0.05). Values without letters indicate that no differences were observed (*p >* 0.05).

**Table 4:** Effect of different proportions of hydroponic barley seedlings replacing oat hay on nitrogen metabolism of heifers.

Items	Treatment			SEM	*p*-Value
	CON	25% HBS	50% HBS		
N intake, g/d	172.80	175.36	173.75	0.78	0.42
Urine volume, L/d	15.34	15.66	18.16	0.57	0.09
N output, g/d					
Feces	63.72 ^c^	68.99 ^b^	78.15 ^a^	1.35	<0.01
Urine	60.42	59.29	58.65	1.39	0.88
Retention ^1^	48.66 ^a^	47.08 ^a^	36.94 ^b^	1.73	0.01
N output, % of N intake					
Feces	37.17 ^b^	39.78^b^	45.16 ^a^	0.77	<0.01
Urine	34.84	33.64	33.74	0.81	0.81
Retention	27.99 ^a^	26.58 ^a^	21.10 ^b^	1.01	0.01

^1^ Nitrogen retention = ingested N − fecal N − urinary N. The values with different small letters in the same row show differences (*p <* 0.05). Values without letters indicate that no differences were observed (*p >* 0.05).

**Table 5:** Effects of different proportions of hydroponic barley seedlings replacing oat hay on the mean proportions (%) on different general in the differential ruminal bacteria of heifers.

Items	Treatment			SEM	*p*-Value
	CON	25% HBS	50% HBS		
*Lachnospiraceae_NK3A20_group*	0.86 ^b^	1.09 ^ab^	1.58 ^a^	0.12	0.02
*Lachnospiraceae_XPB1014_group*	0.73 ^b^	1.09 ^a^	1.25 ^a^	0.08	0.01
*norank_f_Bacteroidales_RF16_group*	1.24 ^a^	0.79 ^b^	0.75 ^b^	0.09	0.03
*unclassified_f_Lachnospiraceae*	0.83 ^b^	0.81 ^b^	1.12 ^a^	0.06	0.04
*SP3-e08*	0.40 ^a^	0.71 ^a^	0.22 ^b^	0.06	<0.01
*unclassified_f_Rikenellaceae*	0.38 ^a^	0.31 ^a^	0.14 ^b^	0.04	<0.01
*norank_f_norank_o_WCHB1-41*	0.35 ^a^	0.24 ^ab^	0.17 ^b^	0.03	0.03
*Sphaerochaeta*	0.24 ^a^	0.20 ^ab^	0.09 ^b^	0.02	<0.01
*Ruminiclostridium*	0.14 ^a^	0.13 ^a^	0.08 ^b^	0.01	<0.01
*Lachnospiraceae_FCS020_group*	0.05 ^ab^	0.04 ^b^	0.08 ^a^	0.01	0.04
*unclassified_o_Oscillospirales*	0.06 ^a^	0.04 ^ab^	0.03 ^b^	<0.01	0.03
*Colidextribacter*	0.01 ^b^	0.04 ^a^	0.08 ^a^	0.01	0.02
*Bacillus*	0.02 ^b^	0.04 ^b^	0.06 ^a^	0.01	<0.01
*norank_f_norank_o_LD1-PB3*	0.01 ^b^	0.04 ^a^	0.02 ^ab^	0.01	0.03
*Agathobacter*	0.01 ^b^	0.02 ^ab^	0.02 ^a^	<0.01	0.03

The values with different small letters in the same row show differences (*p <* 0.05). Values without letters indicate that no differences were observed (*p >* 0.05).

## Data Availability

All sequencing data are available in the NCBI Sequence Read Archive (SRA), under the BioProject ID: PRJNA857201.

## Supplementary Materials

The following supporting information can be downloaded at: https://www.mdpi.com/article/10.3390/microorganisms10102000/s1, Figure S1: Venn diagram illustrating overlap of ruminal microbial operational taxonomic units (OTUs) (A), and Alpha diversity indices of heifers’ ruminal bacteria (B, C, D) between the control and the experimental groups. Figure S2: Growth diagram of hydroponic barley seedlings.
