# Supplementary material for: Partial Replacement of Oat Hay with Whole-Plant Hydroponic Barley Seedlings Modulates Ruminal Microbiota and Affects Growth Performance of Holstein Heifers
Source: Microorganisms. 2022 Oct 10;10(10):2000. doi: 10.3390/microorganisms10102000 (PMC9608837; doi:10.3390/microorganisms10102000)
Supplement: Supplementary file 1 [file microorganisms-10-02000-s001.zip › microorganisms-1902907-supplementary.pdf]

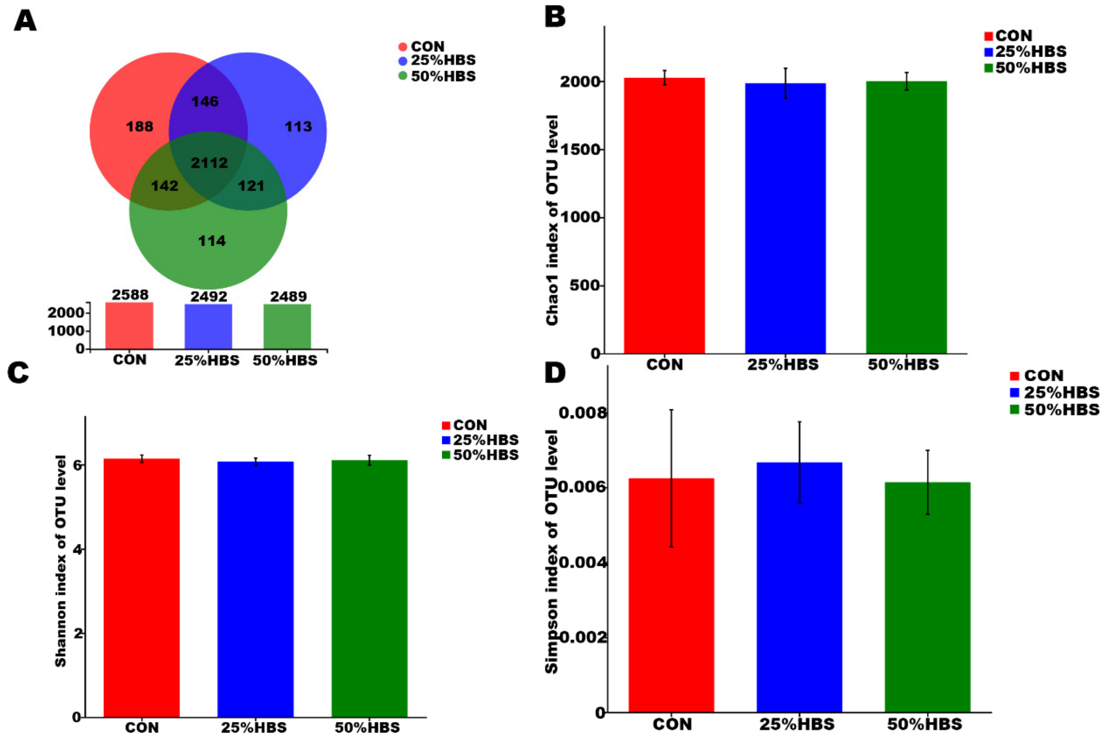

Figure S1. Venn diagram illustrating overlap of ruminal microbial operational taxonomic units (OTUs) (A), and Alpha diversity indices of heifers' ruminal bacteria (B, C, D) between the control and the experimental groups.

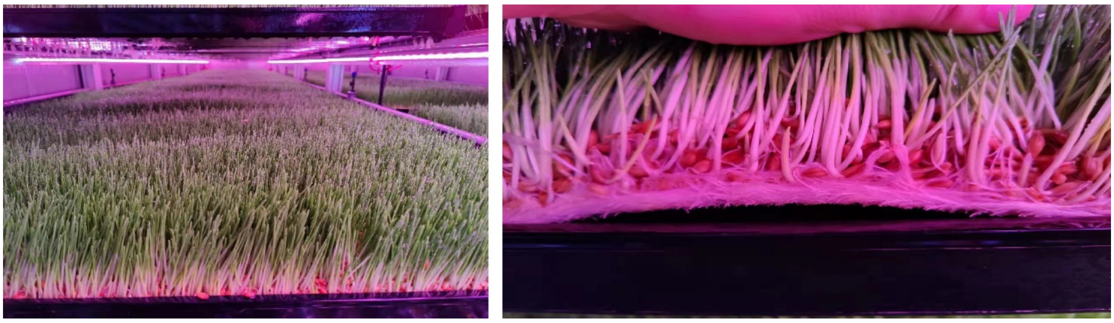

Figure S2. Growth diagram of hydroponic barley seedlings.
